# The Evaluation and Management of Lung Metastases in Patients with Giant Cell Tumors of Bone in the Denosumab Era

**DOI:** 10.3390/curroncol31040160

**Published:** 2024-04-09

**Authors:** Giulia Trovarelli, Arianna Rizzo, Mariachiara Cerchiaro, Elisa Pala, Andrea Angelini, Pietro Ruggieri

**Affiliations:** 1 Department of Orthopedics and Orthopedic Oncology, University of Padua, 35128 Padua, Italy; giulia.trovarelli@aopd.veneto.it (G.T.); ariannarizzo22@gmail.com (A.R.); mariachiara.cerchiaro@unipd.it (M.C.); elisa.pala@unipd.it (E.P.); andrea.angelini@unipd.it (A.A.); 2 Department of Surgery, Oncology and Gastroenterology (DISCOG), University of Padova, 35128 Padua, Italy

**Keywords:** lung metastases, giant cell tumor, bone, treatment, oncologic outcomes

## Abstract

Giant cell tumor of bone (GCTB) is characterized by uncertain biological behavior due to its local aggressiveness and metastasizing potential. In this study, we conducted a meta-analysis of the contemporary literature to evaluate all management strategies for GCTB metastases. A combination of the terms “lung metastases”, “giant cell tumor”, “bone”, “treatment”, and “oncologic outcomes” returned 133 patients meeting our inclusion criteria: 64 males and 69 females, with a median age of 28 years (7–63), at the onset of primary GCTB. Lung metastases typically occur at a mean interval of 26 months (range: 0–143 months) after treatment of the primary site, commonly presenting as multiple and bilateral lesions. Various treatment approaches, including surgery, chemotherapy, radiotherapy, and drug administration, were employed, while 35 patients underwent routine monitoring only. Upon a mean follow-up of about 7 years (range: 1–32 years), 90% of patients were found to be alive, while 10% had died. Death occurred in 25% of patients who had chemotherapy, whereas 96% of those not treated or treated with Denosumab alone were alive at a mean follow-up of 6 years (range: 1–19 years). Given the typically favorable prognosis of lung metastases in patients with GCTB, additional interventions beyond a histological diagnosis confirmation may not be needed. Denosumab, by reducing the progression of the disease, can play a pivotal role in averting or delaying lung failure.

## 1. Introduction

Giant cell tumors of bone (GCTBs) are locally aggressive and rarely metastasizing neoplasms which primarily affect young individuals, accounting for 4–5% of all primary bone tumors [1]. Historically, they were classified as benign tumors. In contrast, they are now considered tumors of “uncertain behavior” due to their local aggressiveness and potential for metastases [1]. GCTB typically presents as a localized tumor causing pain, loss of function, and pathologic fractures in up to 20% of cases [2]. The risk of local recurrence within two years following surgical treatment ranges from 5% to 50%, depending on the type of treatment [3,4]. GCTB may rarely exhibit a multicentric presentation with synchronous or metachronous bone lesions and increased local aggressiveness [5], or it may lead to metastatic lung disease in up to 7% of patients [4,6]. A malignant transformation occurs in about 3% of cases [1,7]. Histologically, GCTB lung metastases resemble the primary lesion and are typically indolent lesions with minimal impact on patient survival [1,8]. However, due to their size and localization, lung metastases can significantly impair pulmonary function, proving lethal [9]. Due to the rarity of the disease, the literature primarily consists of case reports or limited case series, resulting in controversial management approaches. Proposed treatments include follow-up, metastasectomy, radiotherapy, chemotherapy, and Denosumab, without a consensus on superiority [10,11].

The study aims to summarize the available data on the features, biological behavior, treatment, and oncological outcomes of lung metastases in patients with GCTB to provide treatment recommendations.

## 2. Materials and Methods

This review adhered to the PRISMA guidelines [12]. A meta-analysis of the literature was conducted using the PubMed and Google Scholar research libraries to identify all publications since 1980 about the treatment of lung metastases in GCTB. Articles were searched using the following terms and Boolean operators: (Giant cell tumor OR GCT OR BGCT OR GCTB OR (giant cell AND bone)) AND (lung metasta*) AND (treatment OR management OR surg* OR chemo* OR conservative OR denosumab OR bisphosphonates OR adjuvant OR resection OR radio*). Numerous citations were immediately excluded based on information provided by the title or abstract. Additionally, expert opinions, congress proceedings publications, review articles, editorials, letters to the editor, autopsy studies, unpublished case series, and articles containing incomplete or irrelevant information were excluded. The full text of each remaining paper was obtained and assessed against eligibility criteria. The inclusion criteria comprised the following: (1) all manuscripts published between 1980 and 2023 reporting on patients with a confirmed histological diagnosis of lung metastases from GCTB; (2) full-text manuscripts available in English. The exclusion criteria included the following: (1) a lack of complete information with only the abstract available; (2) papers not reporting data on lung metastases in GCTB; (3) an absence of a histological diagnosis of lung metastases of GCTB; (4) a follow-up period shorter than 1 year, unless the patient died of disease; (5) a diagnosis of malignant GCTB.

Two reviewers (G.T. and A.R.) independently double-screened all records for eligibility; a third reviewer (E.P.) checked all excluded records, and a fourth reviewer (A.A.) resolved discrepancies. Additionally, the references of all included studies were scrutinized for additional potentially eligible papers. The methodological quality of the included studies was assessed. When the authors did not specify the level of evidence, two independent reviewers (M.C. and P.R.) assigned levels of evidence to each eligible study. Data extraction was performed by a single individual (A.R.), with independent verification performed by a second reviewer (G.T.). The data extracted from the selected studies into a pre-specified grid included (1) the study design and methodology; (2) patient age and gender and the primary site of the GCTB and its treatment; (3) eventual local recurrence after a primary GCTB and its treatment; (4) the time from initial surgery to onset of lung metastases, their number, and site; (5) the treatment of lung metastases; and (6) the median follow-up period after lung metastases treatment, complications, and oncologic outcomes (local recurrence, disease progression, and patient survival).

Surgical treatments were classified as curettage, wide resection, or amputation, with or without adjuvants (such as phenol, cryotherapy, or polymethylmethacrylate), for primary GCTB and its local recurrence. For lung metastases and their recurrences, surgical treatments included metastasectomy, wedge resection, and lobectomy. Chemotherapy, radiotherapy, drug administration, and observation were considered non-surgical treatments for primary GCTB and lung metastases. Oncologic results were evaluated based on local recurrence, the onset of other metastases, or death; thus, patients were classified as having no evidence of disease (NED), alive with disease (AWD), or dead of disease (DWD). Quantitative variables were summarized using mean and range values. In contrast, qualitative variables were summarized using the number and percentage of patients in each category and compared using Fisher’s exact test.

## 3. Results

**Figure 1:**
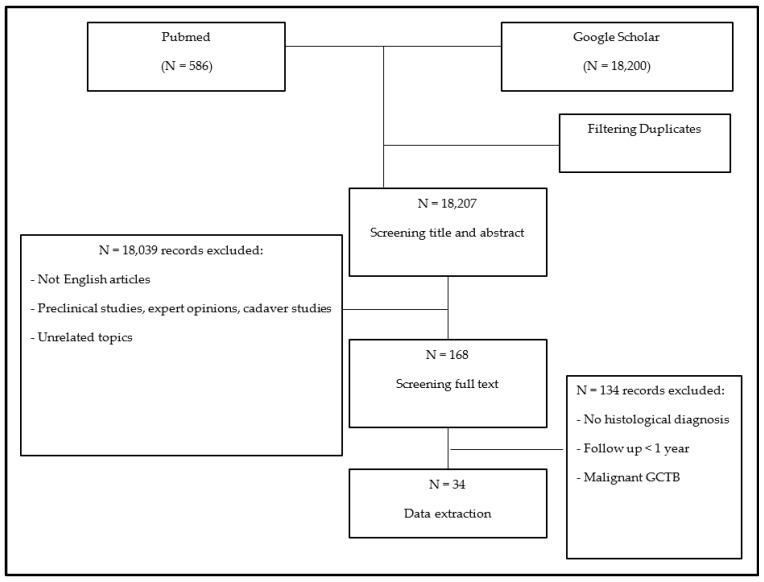
This flowchart shows the methodology applied by the authors during the literature review.

The search strategy yielded 18,786 papers. After removing duplicates and screening titles and abstracts, 168 full-text papers were assessed. Following the application of inclusion and exclusion criteria, 134 records were excluded (Figure 1).

The meta-analysis was conducted on 34 studies classified as evidence level IV, comprising 19 case reports and 15 case series (Table 1).

Initially, 174 patients were included in the analysis. However, 41 patients were subsequently excluded: 29 due to a follow-up period shorter than one year, 9 due to a lack of histological diagnosis, and 3 due to a diagnosis of malignant GCTB.

### 3.1. Features of Patients Included in the Analysis

A total of 133 patients were analyzed: 69 men and 64 women with a mean age of 28 years (range: 7–63 years) at the onset of primary GCTB. The incidence of metastasis in patients with GCTB varied from 2% to 14% in different case series [16,20,23,30,32,33,34,41,45]. The primary site was located around the knee joint in over 50% (69/133) of cases, predominantly in the proximal tibia and distal femur, followed by the axial skeleton (17%, 23/133) and distal radius (14%, 18/133); the most common stage was stage 3 of the Enneking Surgical Staging System (57%, 76/133) [46]. Surgical treatment was performed in almost all cases (95%, 126/133), including curettage eventually associated with adjuvants (66), resection (54), or amputation (7). Local recurrence occurred in 85 cases (64%) at a mean of 22 months (range: 2–108 months) and was primarily managed surgically; in 24% of the cases (20), patients experienced multiple local recurrences. The baseline characteristics of the patients are reported in Table 2.

Lung metastases occurred a mean of 26 months (range: 0–12 years) after the treatment of the primary GCTB, with synchronous presentation in 10% of patients (13/133), and as multiple lesions in 41% (54/133), typically more than 5 (range: 1–99), with bilateral involvement in 81% of cases (44/54) (Table 3).

### 3.2. Treatment of Lung Metastases

The first-line treatment comprised surgery in 68 patients, chemotherapy in 22, radiotherapy in 6, and Denosumab in 13; treatments were combined in 10 patients. Thirty-five patients underwent routine monitoring only.

#### 3.2.1. Surgery

Metastasectomy was the most common surgical approach (39/68, 57%), followed by wedge resection (7/68, 10%) and lobectomy (3/68, 4%). The surgical approach was unspecified in 19 cases (28%) (Table 4).

Occasionally, surgery was combined with Denosumab (two cases), chemotherapy (two cases), radiotherapy (one case), or both radiotherapy and chemotherapy (two cases). The complete removal of all metastatic nodules was achieved in 78% of surgeries, whereas incomplete resection was reported in 15 cases. Among patients with remaining lesions, only one was immediately treated with combined radiotherapy and chemotherapy, whereas fourteen were just observed. Progression was observed after incomplete resection in four cases (4/15, 27%). Treatments for progression included Denosumab (three cases) and surgery followed by chemotherapy (one case). The remaining patients received no further treatments. New lesions following complete surgical resection were identified in 11 cases (11/53, 21%) and were treated with either another surgery (10) or radiotherapy (1). In summary, 15 patients (15/68, 22%) required further treatment after initial surgery, with no significant difference observed between incomplete and complete surgical removal (4/15 and 11/53, *p* = 0.7264).

#### 3.2.2. Chemotherapy and Radiotherapy

Chemotherapy and radiotherapy were the first-line treatments in 23 patients (Table 5), primarily for multiple and bilateral lung lesions, either as an adjuvant (7) or standalone (16) treatment.

The drug regimen varied across studies, with Ifosfamide, Adriamycin, and Cyclophosphamide being the most frequently used drugs. Chemotherapy was combined with radiotherapy in five cases. Radiotherapy alone was administered after surgery in one patient. Disease progression occurred in 33% of patients (8/24), necessitating second-line or third-line chemotherapy (6) or treatment with human recombinant interferon-alpha (1). One patient subsequently experienced spontaneous regression of disease and never needed further treatments.

#### 3.2.3. Denosumab

Denosumab was employed as a first-line treatment for lung metastases in 13 patients (Table 6).

The treatment regimen reported in each study consisted of 120 mg of Denosumab per week with or without a loading dose in the first month. In two patients, radical surgery was planned due to a significant reduction in tumor size after neoadjuvant Denosumab. Disease progression after the first 6 months of treatment occurred in three cases (3/13, 23%), leading to additional treatments such as anti-VEGFR monoclonal antibody therapy (2) or stereotactic radiotherapy (1). No significant adverse effects were reported; only fever and redness occurred in a few cases. The minimum duration of therapy was not reported, and all patients who did not undergo surgery remained under treatment at the time of publication.

#### 3.2.4. Observation

At least observation alone was chosen in 35 patients (Table 7), primarily characterized by multiple and bilateral lung metastases. Most patients (16/35, 47%) reported stable disease without any therapy, and 2 patients even experienced spontaneous regression of the disease. In 15 patients (15/35, 43%), disease progression necessitated surgery (13) or Denosumab (2) treatment; two patients who declined any offered surgical or systemic treatment passed away.

### 3.3. Oncologic Outcomes

The mean follow-up after metastasis diagnosis was 7 years (range: 6 months–32 years). Following our inclusion and exclusion criteria, two patients who succumbed to the disease within 6 months from treatment were included. At the last follow-up, 123 patients (90%) were alive, either without evidence of disease (61) or with stable disease (62), while 10 patients had died. Death was due to the progression of the disease (8/10, 80%) or related to complications of chemotherapy, such as cardiac failure and septic shock (2/10, 20%). Death due to the progression of disease occurred in 25% (6/24) of the chemotherapy group, in 6% (2/35) of the observation group, in 1% of the surgery group (1/68), and in one patient who received combined therapy (surgery and chemotherapy). Patients initially untreated or treated solely with Denosumab showed a 96% survival rate at a mean follow-up of 6 years (range 1–19).

## 4. Discussion

GCTB usually involves the metaphysis and the epiphysis of the long bones, more frequently affecting the distal femur and proximal tibia [1,47,48]. Onset typically occurs in the second to fourth decades of life [1,47,48]. There are no significant differences in occurrence between men and women [49,50]. Histologically, GCTBs comprise mononuclear stromal cells, macrophages, and osteoclast-like giant cells. While stromal cells demonstrate neoplastic and proliferative features, expressing the receptor activator of nuclear factor kappa-B ligand (RANK-L), giant cells are non-neoplastic [1] but constitute the primary tumor component and induce osteolysis, leading to potential cortical bone narrowing and soft tissue expansion [2]. Immunohistochemistry aids in confirming the diagnosis, especially when clinical or morphologic features are inconclusive. Neoplastic cells typically test positive for G34W+ and contain H3F3A mutations [51,52]. Among giant-cell-rich bone tumors, H3G34W immunohistochemistry is highly specific for GCTB and is vital for a differential diagnosis, particularly with giant-cell-rich osteosarcoma [52].

GCTB can manifest as a latent (Stage 1), active (Stage 2), or aggressive (Stage 3) lesion, according to the Enneking classification for benign bone tumors [46]. Stage 1 lesions are delimited by a true capsule visible as a sclerotic rim on X-ray, CT, and MRI. In contrast, Stage 2 lesions lack a true capsule, and, even if they are confined within an anatomical compartment, the cortex may be focally interrupted. Stage 3 lesions extend beyond the compartment of origin with a broken or canceled cortex [46].

GCTB can cause pain and morbidity, leading to joint function loss, with pathological fractures occurring in up to 20% of cases [3]. Local recurrences after surgical treatment are common, ranging from 5% to 50% within two years, depending on the treatment type used [4,7]. Historically classified as a benign tumor, which represents about 20% of cases [1], GCTB is now categorized as a tumor of “uncertain behavior” due to its intrinsic aggressiveness both locally and systemically. Approximately 1% of all GCTBs may have a synchronous or metachronous multicentric presentation with increased local aggressiveness [5,53]. Additionally, up to 3% [1] of lesions may demonstrate malignant characteristics, leading to a worse prognosis and a higher prevalence of sarcomatous pulmonary metastases [54]. Metastases are possible in GCTB even without malignant characteristics, likely due to hematogenous dissemination [7,8].

Typically, metastatic lesions localize in the lungs and histologically resemble the primary lesion [1,4,6]. Lung metastases in GCTB are generally described as indolent, with a longer doubling time than other metastatic lesions [32,55], slow growth, and a favorable prognosis in 70% of cases [9]. However, as they increase in size and localization, lung metastases can significantly compromise pulmonary function and, in rare instances, even prove lethal [56]. Consequently, predicting the biological behavior and progression of the disease is challenging. There is no consensus in the literature regarding risk factors for metastasis development nor a definitive treatment protocol for these lesions. Therefore, we conducted a meta-analysis of the literature to assess patient characteristics, disease biological behavior, and treatment guidelines.

The primary limitation of this study stems from the rarity of the disease. All included studies, primarily case reports, are categorized as level-IV-evidence studies, hindering extensive statistical analysis. Secondly, to ensure homogeneity and avoid potential confounding data, we excluded patients without a confirmed histological diagnosis or with a follow-up shorter than 1 year after treatment. Unfortunately, in some studies in which patients did not undergo surgical treatment, biopsies were not performed, resulting in their exclusion from this analysis and reducing the number of non-surgically treated cases. While narrowing the focus may introduce selection bias, we believe the effective histological diagnosis of lung metastasis, excluding metastases from other carcinomas, sarcomas, or malignant GCTB, is crucial for better understanding the disease biology and determining an appropriate treatment. Since 2013, the utilization of H3G34W mutation analysis has emerged as a method of validating the histological diagnosis of GCTB [57]. Regrettably, most of the articles reviewed in this study were published before 2013 and therefore did not include this somatic driver mutation in their analysis. Thirdly, the treatment groups lacked uniformity, potentially influencing treatment choices based on disease burden or perceived aggressiveness and introducing bias. Patients with more severe conditions may have been offered more aggressive treatments, leading to more complications and poorer outcomes.

Our analysis suggests that lung metastases in GCTB are more prevalent than previously reported (2–4%) [15,20], occurring in approximately 7–10% of patients [16,22,23,32,33,34,41] and up to 14% in spinal GCTB cases [16]. Our findings align with the literature [58], indicating that most metastases are multiple and bilateral, predominantly located in the lung periphery. Synchronous cases constitute only 10%, with metastases more commonly appearing during follow-up, usually within 3 years post primary lesion treatment [58].

The literature lacks consensus on risk factors for developing lung metastases concerning sex, age, site, stage, and primary GCTB treatment type. Previous reports suggested no association between age, sex, and metastatic lesions, with a median age similar to that generally reported for GCTB [1,32,55]. Similarly, we observed no significant gender differences. However, although the median age at diagnosis in our case series was 28 years, consistent with primary GCTB, 70% of patients who developed lung metastases were younger than 30 at primary lesion onset [4,7,10,23,50,56,58,59]. Some authors proposed that the primary location may not predict metastases, with studies showing no significant differences in primary GCTB site distribution between patients with and without lung metastases [58,59]. Yang et al. [59] found no significant difference matching the sites of primary GCTB between patients who developed lung metastases and the general GCTB population. Accordingly, Rosario et al. [58], in their surveillance study on 333 patients, reported that the primary tumor site is not linked with the risk of metastatic development. However, others identified the spine site as a risk factor for lung metastases [16,60]. Donthineni et al. [16] reported a 14% incidence of lung metastases in patients with primary GCTB localized in the spine. Similar results were reported by Chan et al.: axial localization was observed in 27% of metastatic cases, compared to only 6% of non-metastatic ones [60]. In our case series, the primary site was around the knee joint in more than 50% of patients, consistent with the most frequent sites of GCTB. However, axial localization was prevalent in our case series, mainly in the spine, suggesting it as an additional risk factor. Moreover, Enneking Stage 3 GCTB, indicative of an extracompartmental lesion, has been noted as a primary metastasis risk factor due to its aggressive nature [20,22,60]. In their case series, Chan et al. [60] and Dominkus et al. [20] found that 100% of the metastatic patients were in Enneking stage 3. Furthermore, Yang et al. [59] found a significant difference in Enneking stage 3 patients in the non-metastatic group (32%) and in the metastatic group (100%). On the contrary, Rosario et al. [58] and Tsukamoto et al. [23] did not report significant differences between the same groups. Moreover, some studies [14,16,34,50,59,60] report the type of primary surgery (curettage vs. resection vs. amputation) as an intrinsic risk factor for the outbreak of metastatic lesions. Yang et al. [59] reported a significant difference in the type of primary surgery between metastatic and non-metastatic groups, with the prevalence of curettage significantly higher in the metastatic group (80% vs. 55%). However, pulmonary lesions may be detected simultaneously or before the primary lesion [25,35,36]; thus, the type of surgery should not be considered a risk factor. In our case series, 57% of cases were in Enneking stage 3, with curettage being the most common primary bony lesion treatment. However, our data did not clarify its association with metastasis development. Nevertheless, an association between local recurrence and metastatic lesion development was observed in the literature [20,22,36,45,50,56,58,59]. For instance, Yang et al. [59] reported a recurrence rate of 74% in a metastatic group and 12% in a non-metastatic one. Rosario et al. [58] found that metastatic lesions occur in 36% of patients with recurrence. In our case series, 64% of patients had a previous local recurrence, with 23% experiencing multiple recurrences.

Our analysis suggests that the combination of these risk factors can likely increase the possibility of metastases. The primary risk factor for developing lung metastases appears to be patients younger than 30 with aggressive stage 3 GCTB experiencing one or more recurrences after surgical treatment. Axial localization seems to be an additional risk factor. Consequently, careful periodic surveillance with thoracic CT after diagnosing GCTB should be recommended, particularly in case of bone recurrence.

The treatment choice for metastatic lesions remains a controversial topic in the current literature. Older articles recommended prompt surgical intervention as it effectively prevents pulmonary dysfunction [34,36,61]. However, lesions often remain stable, and spontaneous regression is observed in over 4% of cases [10]. Therefore, the recent literature tends to be more conservative, suggesting surgical treatment only after evidence of progression [33,45,58].

Various types of surgeries can be performed, such as metastasectomy, wedge resection, or lobectomy, resulting in the complete or incomplete removal of all lesions [10]. Our analysis demonstrates excellent oncological outcomes associated with surgical procedures, with only 2% of patients dead of disease. Interestingly, similar disease progression rates and a need for further treatments were observed regardless of the aggressiveness of the surgery. Moreover, most remaining lesions remained stable during follow-up. Thus, choosing a less aggressive surgery appears reasonable to avoid morbidity without compromising survival.

Historically, chemotherapy and radiotherapy were frequently utilized, particularly in cases of multiple and bilateral lung lesions [10]. However, the efficacy of chemotherapy in GCTB has been limited, primarily due to variations in drug selection, without a gold standard. Conversely, the associated side effects are well recognized [10,29]. Our analysis indicates that patients treated with chemotherapy as a first-line treatment experienced disease progression in 33% of cases, with lethal complications observed in 8%, suggesting a significant reduction in its use in patients with GCTB lung metastases. Conventional radiotherapy has been employed for decades [17]. However, few studies have reported favorable clinical outcomes following the use of stereotactic therapy for primary GCTB [62,63,64] and lung metastasis [17]. Nonetheless, radiotherapy carries a risk of up to 5% of post-radiation sarcomas [65], posing a significant concern, particularly in young patients with indolent disease. Consequently, its use has been limited due to potential long-term complications.

Denosumab was introduced and progressively replaced chemotherapy or radiotherapy for unresectable lesions. It is a fully human monoclonal antibody that targets the highly expressed RANK-L, inhibiting the recruitment of reactive osteoclast-like giant cells and thus preventing osteolysis [7]. Denosumab therapy effectively reduces the number of osteoclastic cells in GCTB. However, the neoplastic cells, which are G34W+ and contain H3F3A mutations, survive [51], eventually requiring surgical treatment upon Denosumab cessation. Recent studies [10,56] have demonstrated the efficacy of this treatment in reducing tumor mass and pain. However, it is associated with side effects, such as hypocalcemia and hypercholesterolemia [10,56]. Serious side effects, including osteonecrosis of the jaw, atypical fracture, and sarcomatous degeneration, are, fortunately, uncommon [66,67]. None of the patients in our analysis experienced disease progression or required second-line treatment. Furthermore, positive outcomes are associated with lower morbidity than surgical treatment [67]. Despite 23% of patients experiencing disease progression in the first 6 months of treatment, all patients treated with Denosumab were alive at the last follow-up without remarkable side effects. The treatment regimen has been standardized, including 120 mg weekly after a loading dose. However, the suggested duration of the therapy remains undetermined. Denosumab can also be associated with anti-VEGF monoclonal antibodies in cases of poor response or specific mutations [19,29]. However, only three cases of anti-VEGF treatment for GCTB lung metastases are reported in the literature [19,29,61]. While the outcomes were positive, more data are required to confirm the possibility of enhancing the clinical response to Denosumab. IFN has also been used in lung nodules refractory to chemotherapy [68], demonstrating positive clinical outcomes with no evidence of disease progression. However, the suggested dosage remains unclear, and severe reported side effects include depression and ischemic events [23].

A wait-and-see approach is considered a viable option for non-symptomatic and stable lesions given the high rates of spontaneous regression or stable disease observed during long-term follow-up [10,56]. In a study conducted in 2020, Tsukamoto et al. [23] reviewed 22 patients initially managed with observation and found that disease progression occurred in only 54% of cases, primarily in lesions larger than 5 mm. In contrast, less than 45% of patients with nodules smaller than 5 mm experienced progression, indicating that surveillance alone was sufficient. Our study reveals that disease progression may occur in more than 43% of patients, necessitating second-line treatment; however, with interventions such as surgery or Denosumab, survival was not affected except in two patients who declined treatment and subsequently passed away.

In summary, based on the studies mentioned, observation can be considered a viable option, with Denosumab being the preferred initial choice and surgery reserved for cases of progression.

## 5. Conclusions and Future Directions

In conclusion, lung metastases are not uncommon in GCTB. They are synchronous with primary lesions in only 10% of cases; more frequently, they can occur many years after treatment. Therefore, careful periodic surveillance with thoracic CT after diagnosing GCTB is recommended, especially in patients younger than 30 with an aggressive stage 3 GCTB who have experienced one or more local recurrences. Typically, lung metastases are multiple and bilateral, with a good prognosis. Thus, once the histological diagnosis has been confirmed, a wait-and-see approach should be favorable, considering the high rates of spontaneous regression or stable disease during long-term follow-up. Metastasectomy should be reserved for single or few actively growing lesions. Denosumab, by reducing the progression of the disease, can help avoid surgery and prevent lung failure.

## Figures and Tables

**Table 1:** Selected papers (n = 34).

Authors	N of Included Patients	Authors	N of Included Patients
De Fazio et al., 2023 [13]	Case report	Moon et al., 2012 [14]	Case report
Leland et al., 2023 [8]	Case report	Jacopin et al., 2010 [9]	Case report
Miwa et al., 2023 [15]	Case report	Donthineni et al., 2009 [16]	6
Feng et al., 2022 [17]	Case report	Ropars et al., 2017 [18]	Case report
Gong et al., 2021 [19]	Case report	Dominkus et al., 2006 [20]	13
Orr et al., 2020 [21]	Case report	Faisham et al., 2006 [22]	4
Tsukamoto et al., 2020 [23]	22	Osaka et al., 2004 [24]	5
Dury et al., 2019 [25]	Case report	Fadare et al., 2002 [26]	Case report
Reddy et al., 2021 [27]	Case report	Feigenberg et al., 2002 [28]	2
Wang et al., 2019 [29]	Case report	Sanjay et al., 1998 [30]	3
Luo et al., 2018 [31]	7	Takanami et al., 1998 [32]	4
Kito et al., 2017 [33]	12	Kay et al., 1994 [34]	6
Yamagashi et al., 2016 [35]	Case report	Siebenrock et al., 1998 [36]	20
Carvalho de Medeiros et al., 2011 [37]	Case report	Kaiser et al., 1993 [38]	Case report
Naam et al.,2014 [39]	Case report	Ladanyi et al., 1989 [40]	4
Liu et al., 2013 [41]	4	Mirra et al., 1982 [42]	Case report
Hamann et al., 2012 [43]	Case report	Vanel et al., 1983 [44]	2

**Table 2:** Characteristics of patients at the initial presentation of GCTB (n = 133).

Characteristic	N (%)
*Sex*	
Female	64 (48)
Male	69 (52)
*Age (Years)*	28.2 ± 10.7 (IC: 7–63)
*Site*	
Proximal humerus	4 (3)
Distal humerus	4 (3)
Proximal radius	1 (1)
Distal radius	18 (13)
Distal ulna	1 (1)
Hand (metacarpal, phalanx)	10 (7)
Proximal femur	1 (1)
Distal femur	26 (19)
Patella	5 (4)
Proximal tibia	31 (23)
Proximal fibula	7 (5)
Distal tibia	2 (1)
Vertebrae	12 (9)
Sacrum	6 (4)
Pelvis	5 (4)
*Stage (according to Enneking classification for benign tumors)*	
Unknown	41 (31)
2	16 (12)
3	76 (57)
*Treatment of primary bone lesion*	
Curettage ± adjuvant	66 (50)
Resection	54 (41)
Amputation	7 (5)
Observation	1 (1)
Radiotherapy ± chemotherapy	1 (1)
Unknown	4 (3)
*Local recurrence*	
Yes	85 (64)
No	44 (33)
Unknown	4 (3)
Multiple recurrences	20 (15)
*Time to recurrence*	22.3 ± 22.2 (IC: 2–108)

**Table 3:** Characteristics of metastatic lesions (n = 133).

Characteristic	N (%)
*Location of metastases*	
Unilateral	7 (5)
Bilateral	44 (33)
Unknown	82 (59)
*Time to metastases (months)*	26.1 ± 27 (IC: 0–143)
*First-line treatment*	
Surgery alone	61 (46)
Observation alone	35 (26)
Chemotherapy alone	16 (12)
Denosumab alone	11 (8)
Radiotherapy alone	0 (0)
Combined treatments	10 (7)
Chemotherapy and radiotherapy	3
Surgery and Denosumab	2
Surgery and chemotherapy	2
Surgery and radiotherapy	1
Surgery, chemotherapy, and radiotherapy	2
*Follow-up (months)*	85 ± 76 (IC: 6–374)
*Progression of disease*	41 (31)
After surgery	15 (22)
After radiotherapy	0 (0)
After chemotherapy	8 (35)
After Denosumab	3 (23)
After observation	15 (43)
*Second-line treatment*	
Surgery	23 (56)
Radiotherapy	2 (5)
Chemotherapy	4 (10)
Denosumab	5 (12)
Denosumab + antiVEGFR	2 (5)
Interferon alpha	1 (2)
Observation	4 (10)
*Outcomes*	
No evidence of disease	61 (46)
Alive with disease	62 (47)
Dead of disease	10 (7)

**Table 4:** Surgical treatment group (n = 68).

Characteristic	N (%)
*Location of metastases*	
Unilateral	4 (6)
Bilateral	14 (20)
Unknown	50 (73)
*Time to metastases (months)*	32.2 ± 24.2 (IC: 0–143)
*Follow-up (months)*	94.2 ± 74.9 (IC: 12–384)
*Type of resection*	
Complete resection	53 (78)
Partial resection	15 (22)
*Type of surgery*	
Metastasectomy	39 (57)
Wedge resection	7 (10)
Lobectomy	3 (4)
Unknown	19 (28)
*Progression of disease*	15 (22)
After partial resection	4
After complete resection	11
*Second-line treatment*	
Surgery	10
Denosumab	3
Radiotherapy	1
Surgery and chemotherapy	1
*Outcomes*	
No evidence of disease	52 (76)
Alive with disease	14 (21)
Dead of disease	2 (3)

**Table 5:** Systemic therapy group (n = 23).

Characteristic	N (%)
*Location of metastases*	
Unilateral	1 (4)
Bilateral	9 (36)
Unknown	14 (40)
*Time to metastases (months)*	22.5 ± 28.5 (IC: 0–117)
*Follow-up (months)*	72.5 ± 77 (IC: 6–264)
*Type of treatment*	
Chemotherapy	16 (70)
Chemotherapy and radiotherapy	3 (13)
Chemotherapy, radiotherapy, and surgery	2 (9)
Chemotherapy and surgery	1 (4)
Radiotherapy and surgery	1 (4)
*Progression of disease*	8 (33)
*Second-line treatment*	
Chemotherapy	6
Interferon-alpha	1
Observation	1
*Outcomes*	
No evidence of disease	4 (17)
Alive with disease	13 (52)
Dead of disease or side effects of the therapy	7 (29)

**Table 6:** Denosumab group (n = 13).

Characteristic	N (%)
*Number and location*	
Unilateral	1 (6)
Bilateral	6 (46)
Unknown	6 (46)
*Time to metastases (months)*	27.8 ± 27.2 (IC: 0–60)
*Follow-up (months)*	26.8 ± 10.3 (IC: 15–48)
*Progression*	3 (23)
*Second-line therapy*	
Denosumab + anti-VEGFR monoclonal antibodies	2
Stereotactic therapy	1
*Outcome*	
No evidence of disease	2 (15)
Alive with disease	11 (85)
Dead of disease	0

**Table 7:** Observation group (n = 35).

Characteristic	N (%)
*Location of metastases*	
Unilateral	2 (6)
Bilateral	15 (43)
Unknown	18 (51)
*Time to metastases (months)*	20.7 ± 17.5 (IC: 0–60)
*Follow-up (months)*	101.1 ± 74.8 (IC: 6–374)
*Progression of disease*	17 (49)
*Second-line treatment*	
Surgery	13
Denosumab	2
Observation	2
*Outcomes*	
No evidence of disease	9 (26)
Alive with disease	24 (69)
Dead of disease	2 (7)
